# Relationship Between Hypoglycemia Frequency and Academic Performance in Children With Type 1 Diabetes

**DOI:** 10.7759/cureus.107271

**Published:** 2026-04-17

**Authors:** Kiramat U Wazir, Hafsah Naz, Amna Mahmood, Perwez Ali, Ahmad Yar, Nida Imdad, Kaushalendra Mani Tripathi, Abdul Ahad Mehboob

**Affiliations:** 1 Paediatrics, Mardan Medical Complex, Mardan, PAK; 2 Paediatric Medicine, Bakhtawar Amin Memorial Hospital, Multan, PAK; 3 Paediatric Medicine, Children Hospital Lahore, Lahore, PAK; 4 Paediatrics, Bakhtawar Amin Medical & Dental College, Multan, PAK; 5 Paediatric Medicine, University of Child Health Sciences, The Children's Hospital, Lahore, PAK; 6 Paediatrics, Services Hospital, Lahore, PAK; 7 Internal Medicine, White River Health Medical Center, Batesville, USA; 8 Cancer Screening, Royal Surrey NHS Foundation Trust, Surrey, GBR

**Keywords:** academic performance, glycemic control, hypoglycemia, school attendance, type 1 diabetes mellitus (t1dm)

## Abstract

Background: Type 1 diabetes mellitus (T1DM) in children necessitates intensive insulin therapy, which predisposes patients to recurrent hypoglycemic episodes.

Objective: To evaluate the association between the frequency of hypoglycemic episodes and academic performance among children with type 1 diabetes by correlating glycemic patterns with scholastic outcomes.

Methods: This cross-sectional analytical study was conducted at Children Hospital Lahore from March 2024 to March 2025 and included 275 children diagnosed with T1DM. Data regarding hypoglycemic episodes, glycemic control, academic scores, and school attendance were collected and analyzed.

Results: The mean age of participants was 11.8 ± 2.9 years, with a mean glycated hemoglobin (HbA1c) of 8.7 ± 1.4%. The average frequency of hypoglycemic episodes was 5.8 ± 3.4 per month. High-frequency hypoglycemia was observed in 29.1% of children, while 25.8% reported severe episodes. The mean academic score was 74.6 ± 10.8%, and average attendance was 88.5 ± 7.6%. A significant decline in academic performance was noted with increasing hypoglycemia frequency; children in the high-frequency group had lower academic scores (65.8 ± 9.7% vs 82.1 ± 7.6%), reduced attendance (81.7 ± 7.8% vs 93.4 ± 4.2%), and higher absenteeism (21.4 ± 6.2 vs 8.7 ± 3.5 days/year) (p < 0.001). A strong negative correlation was observed between hypoglycemia frequency and academic scores (r = −0.62).

Conclusion: Recurrent hypoglycemia is significantly associated with poorer academic performance and increased absenteeism among children with type 1 diabetes, highlighting the importance of optimizing glycemic control to improve both clinical and educational outcomes.

## Introduction

Type 1 diabetes mellitus (T1DM) is a chronic endocrine disorder in children characterized by autoimmune destruction of pancreatic β-cells, necessitating lifelong insulin therapy. The incidence of T1DM is rising globally at an alarming rate, contributing not only to increasing disease burden but also to significant psychosocial stress among affected children and their families [1]. While insulin therapy plays a crucial role in achieving optimal glycemic control and reducing long-term complications, it is also associated with an increased risk of hypoglycemia, which remains the most common and feared acute complication of diabetes management [2]. The predation of blood glucose that is less than 70 mg/dL is known as hypoglycemia, which is characterized by autonomic and neuroglycopenic symptoms and includes sweating, confusion, poor concentration, seizures, and unconsciousness [3]. The glucose factor is highly dependent on the process of brain development in children, and, therefore, frequent cases may adversely affect cognitive processes, memory, and attention [4]. Experimental and clinical findings show that chronic hypoglycemia has the potential to disrupt the abilities of the hippocampus and neurocognitive development and result in persistent learning problems in the long-term [5]. School-aged children are also subjected academic settings for a large portion of their time, which require sustained attention, processing speed, and executive processing. Even minor instances of hypoglycemia in school may complicate concentration, slow down thinking, and deteriorate performance in the classroom [6]. It might also result in mental stress, school absenteeism, and hospital visits due to extreme cases, thus further reducing performance in school [7]. Subsequently, glycemic variability -particularly frequent hypoglycemic episodes - is closely associated with day-to-day school functioning, exerting a greater impact than overall glycemic control alone [8].

Some researches have also indicated negative correlation between frequent incidences of hypoglycemia and poor memory, attention, and academic testing outcomes [9]. Acute hypoglycemia, and especially the one that happens at earlier stages of life, has been associated with a reduction in IQ and neurocognitive impairments over time [10]. Along with physiological outcomes, phobia of hypoglycemia, the cost of treatment, and constant disruptions to check the level of glucose in the blood could reduce the attendance in classes and self-confidence, which in turn affects the academic performance [11]. Insufficient knowledge among teachers and caregivers may lead to delays in managing hypoglycemic episodes, thereby contributing to disruptions in learning [12,13]. Despite the problems associated with hypoglycemia, not much evidence directly points to the frequency of hypoglycemia having a direct relationship with the measurable variables of academic achievement, e.g., school grades, and attendance [14].

Objective

To evaluate the association between the frequency of hypoglycemic episodes in children with T1DM and their academic performance, defined as examination scores, attendance rates, and classroom functioning; and to analyze this relationship through group comparisons, correlation, and multivariate regression while assessing the influence of glycemic control (glycated hemoglobin (HbA1c)) and other clinical factors.

## Materials and methods

This was a cross-sectional analytical study conducted at Children Hospital Lahore from March 2024 to March 2025, including 275 children diagnosed with T1DM. The study aimed to evaluate the relationship between the frequency of hypoglycemic episodes and academic performance by comparing glycemic event patterns with scholastic outcomes. Eligible patients attending routine pediatric endocrinology outpatient clinics were assessed systematically using standardized clinical and academic evaluation tools. Children eligible for inclusion in the study were aged 6 to 16 years and had a confirmed diagnosis of T1DM for at least one year. All participants were required to be on regular insulin therapy, with routine home glucose monitoring, and to be enrolled in formal schooling during the study period. Availability of academic records or official examination results was mandatory for the assessment of educational performance. In addition, both patients and their parents were required to provide informed consent prior to participation. Children were excluded if they had been newly diagnosed with diabetes (less than one year since diagnosis). Those with chronic systemic illnesses known to affect cognitive function, such as epilepsy, hypothyroidism, or chronic kidney disease, were not included. Participants with known neurodevelopmental or psychiatric disorders, a history of severe head injury or other neurological diseases, or those taking medications that could influence cognitive performance were also excluded. Cases with inconsistent or unverifiable academic or glycemic records were excluded from the final analysis.

Data collection

Data were collected using a structured proforma. Demographic factors were age, gender and years of diabetes. The clinical data included insulin regimen, HbA1c levels, frequency of self-monitoring blood glucose, and monthly incidences of hypoglycemic episodes (mild, self-treated, moderate (need assistance), and severe (need medical help)). Academic performance measures were recent examination scores, the overall grade percentage, attendance rate, and classroom concentration and participation as reported by the teachers. The frequency of hypoglycemia depending on the number of episodes per month was divided into low-, moderate-, and high-frequency categories to be assessed comparatively. Medical records, glucose logs, and confirmed school reports were used to gather all the data so as to achieve reliability and consistency.

Statistical analysis

Data were analyzed using Statistical Package for the Social Sciences (SPSS) version 26. Quantitative variables, such as age, duration of diabetes, HbA1c, hypoglycemia frequency, and academic scores, were expressed as mean ± SD, while categorical variables were summarized as frequencies and percentages. Differences in academic performance among hypoglycemia frequency groups were evaluated using independent sample t-test or one-way analysis of variance (ANOVA) for continuous variables and Chi-square test for categorical variables. A p-value ≤ 0.05 was considered statistically significant.

## Results

Data were collected from 275 participants, the mean age was 11.8 ± 2.9 years, with male predominance (54.2%). The average disease duration was 4.6 ± 2.1 years, most commonly between four and six years (39.6%). Mean HbA1c was 8.7 ± 1.4%, and 28.4% had poor glycemic control (>9%). The majority were managed with multiple daily insulin injections (71.3%), while 28.7% used insulin pumps (Table 1).

**Table 1:** Baseline demographic and clinical characteristics of children with T1DM (n = 275) Data presented as mean ± SD or n (%). HbA1c: Glycated hemoglobin; SMBG: Self-monitoring of blood glucose; T1DM: Type 1 diabetes mellitus

Variable	Category	n (%) (Mean ± SD)
Age (years)	—	11.8 ± 2.9
	6–10 years	104 (37.8)
	11–13 years	96 (34.9)
	14–16 years	75 (27.3)
Gender	Male	149 (54.2)
	Female	126 (45.8)
Duration of diabetes (years)	—	4.6 ± 2.1
	1–3 years	92 (33.5)
	4–6 years	109 (39.6)
	>6 years	74 (26.9)
HbA1c (%)	—	8.7 ± 1.4
	≤8%	101 (36.7)
	8.1–9%	96 (34.9)
	>9%	78 (28.4)
Insulin therapy	Multiple daily injections	196 (71.3)
	Insulin pump	79 (28.7)
SMBG frequency	≤3/day	86 (31.3)
	4–5/day	113 (41.1)
	>5/day	76 (27.6)
Family history	Present	82 (29.8)

Hypoglycemia was frequent, with one-third experiencing low burden, 37.8% moderate burden, and 29.1% high burden, corresponding to 2.1 ± 0.8, 4.6 ± 0.9, and 9.2 ± 2.3 episodes per month, respectively. Mild episodes occurred in all participants, while moderate and severe events affected 68.4% and 25.8%. Nocturnal and school-time hypoglycemia was reported in 42.9% and 59.6% of children, respectively. Impaired awareness was present in 15.3%, and 22.9% required hospitalization, indicating a substantial clinical burden of recurrent hypoglycemia (Table 2).

**Table 2:** Hypoglycemia frequency, severity, and clinical pattern Continuous variables expressed as mean ± SD; categorical variables as n (%).

Variable	Category	n (%)	Episodes/Month (Mean ± SD)
Hypoglycemia burden	Low (<3)	91 (33.1)	2.1 ± 0.8
	Moderate (3–6)	104 (37.8)	4.6 ± 0.9
	High (>6)	80 (29.1)	9.2 ± 2.3
Mild episodes	Present	275 (100)	4.1 ± 2.6
Moderate episodes	Present	188 (68.4)	1.3 ± 1.2
Severe episodes	Present	71 (25.8)	0.4 ± 0.7
Nocturnal hypoglycemia	Yes	118 (42.9)	—
School-time hypoglycemia	Yes	164 (59.6)	—
Impaired awareness	Yes	42 (15.3)	—
Hospitalization	Yes	63 (22.9)	—

Academic performance overall was moderate, with a mean score of 74.6 ± 10.8%. Good or excellent grades were achieved by 65.4% of students, whereas 6.2% had poor performance. Subject-wise means were 72.3 ± 12.4% in mathematics, 76.1 ± 11.2% in language, and 73.9 ± 11.9% in science. Attendance averaged 88.5 ± 7.6%, although over one-third exhibited poor concentration (35.3%) and one-quarter required academic support (25.1%), suggesting notable functional and scholastic challenges (Table 3).

**Table 3:** Academic performance and school functioning Continuous variables expressed as mean ± SD; categorical variables as n (%).

Parameter	Category	n (%) (Mean ± SD)
Overall score (%)	—	74.6 ± 10.8
	Excellent (≥85)	62 (22.5%)
	Good (70–84)	118 (42.9%)
	Average (50–69)	78 (28.4%)
	Poor (<50)	17 (6.2%)
Mathematics score (%)	—	72.3 ± 12.4
Language score (%)	—	76.1 ± 11.2
Science score (%)	—	73.9 ± 11.9
Attendance (%)	—	88.5 ± 7.6
Poor concentration	Yes	97 (35.3%)
	No	178 (64.7%)
Academic support needed	Yes	69 (25.1%)
	No	206 (74.9%)

Children in the high-frequency group demonstrated higher HbA1c levels (9.6 ± 1.3%), lower overall scores (65.8 ± 9.7%), poorer subject performance, reduced attendance (81.7 ± 7.8%), and greater school absences (21.4 ± 6.2 days) compared to the low-frequency group, which showed better glycemic control and higher scores (82.1 ± 7.6%). Poor concentration increased markedly from 15.4% to 56.3% across groups. All differences were statistically significant (p < 0.001) (Table 4).

**Table 4:** Academic outcomes by hypoglycemia frequency group Continuous variables were analyzed using one-way ANOVA to compare mean values across the three hypoglycemia frequency groups. Categorical variables were analyzed using the Chi-square (χ²) test. Data are presented as mean ± SD for continuous variables and number (%) for categorical variables. A p-value <0.05 was considered statistically significant. HbA1C: Glycated hemoglobin; ANOVA: Analysis of variance

Variable	Low (n=91)	Moderate (n=104)	High (n=80)	Test Statistic	p-value
HbA1c (%)	7.9 ± 0.9	8.6 ± 1.1	9.6 ± 1.3	F(2,272)=96.41	<0.001
Overall score (%)	82.1 ± 7.6	74.9 ± 8.4	65.8 ± 9.7	F(2,272)=118.27	<0.001
Mathematics (%)	80.4 ± 8.9	72.6 ± 9.8	61.5 ± 11.3	F(2,272)=102.56	<0.001
Language (%)	83.5 ± 7.2	76.3 ± 8.7	68.9 ± 10.1	F(2,272)=94.33	<0.001
Science (%)	81.6 ± 8.3	73.5 ± 9.5	64.7 ± 11.4	F(2,272)=97.81	<0.001
Attendance (%)	93.4 ± 4.2	88.1 ± 5.6	81.7 ± 7.8	F(2,272)=124.19	<0.001
Absence (days/year)	8.7 ± 3.5	13.9 ± 4.7	21.4 ± 6.2	F(2,272)=176.52	<0.001
Poor concentration, n (%)	14 (15.4)	38 (36.5)	45 (56.3)	χ²(2)=41.63	<0.001

Correlation analysis showed strong negative relationships between hypoglycemia frequency and academic performance (r = −0.62), HbA1c (r = −0.49), and school absence (r = −0.55) (all p < 0.001). Multivariate regression identified high hypoglycemia burden (odds ratio (OR) 3.84), severe hypoglycemia (OR 2.73), and HbA1c >9% (OR 2.41) as independent predictors of poor academic performance, whereas frequent glucose monitoring (>5/day) was protective (OR 0.54) (Table 5).

**Table 5:** Correlation and multivariate predictors of poor academic performance (<70%) Associations between continuous variables were evaluated using the Pearson correlation test (r). Independent predictors of poor academic performance (<70%) were identified using multivariate logistic regression analysis, and results are presented as ORs with 95% CIs. A p-value <0.05 was considered statistically significant. HbA1c: Glycated hemoglobin; SMBG: Self-monitoring of blood glucose; CI: Confidence interval; OR: Odds ratio

Variable	Effect Size	95% CI	p-value
Hypoglycemia frequency	−0.62	−0.70 to −0.53	<0.001
HbA1c	−0.49	−0.58 to −0.39	<0.001
School absence	−0.55	−0.64 to −0.45	<0.001
Severe hypoglycemia	2.73	1.48–5.02	0.001
High hypoglycemia group	3.84	2.11–6.98	<0.001
HbA1c >9%	2.41	1.36–4.27	0.002
SMBG >5/day	0.54	0.31–0.93	0.027
Insulin pump therapy	0.68	0.39–1.17	0.160

## Discussion

This study analyzed 275 children with T1DM who demonstrated moderate overall glycemic control (HbA1c 8.7 ± 1.4%). However, glycemic stability remained suboptimal, with a mean of 5.8 ± 3.4 hypoglycemic episodes per month; 29.1% of patients experienced high-frequency episodes, and 25.8% reported severe hypoglycemic events. Recurrent and treatment-associated hypoglycemia has been documented to be comparable in earlier investigations, which also observed that the intensive insulin treatment usually exposes children to recurrent and nocturnal events [15]. Hypoglycemia episodes during school time and nocturnal periods were observed in 59.6% and 42.9% of the subjects, respectively, suggesting that a significant number of events occurred during cognitively demanding or vulnerable periods. Earlier research also found that daytime and nocturnal hypoglycemia are common occurrences among pediatric patients and are associated with poor attention and learning performance the next day [16]. Our group performance was mediocre, with a mean score of 74.605±10.885 and a mean attendance of 88.5±7.6. Previous studies have also reported similar performance drop in the classroom as well as increased learning problems in children with diabetes, and glycemic variability has been associated with reduced school engagement [17].

The dose response was evident in the groups of hypoglycemia. Children with low-frequency episodes scored higher (82.1%, 7.6), and children with high-frequency episodes scored lower (65.8%, 9.7). Children with low-frequency episodes had better attendance (93.4%, 4.2), and those with high-frequency episodes had poor attendance (81.7%, 7.8). This is consistent with previous literature that has already shown a negative association between increasing hypoglycemia frequency and educational attainment [18]. There was no correlation analysis showing that hypoglycemia frequency was most negatively correlated with academic scores (r = -0.62), followed by HbA1c (r = -0.49). Other prior investigations also emphasized that glucose variability and acute hypoglycemia appear to be stronger correlates of cognitive dysfunction [19]. Multivariate analysis was also used to confirm that high-frequency hypoglycemia was a strong independent predictor (OR 3.84) and that frequent glucose monitoring was protective (OR 0.54), though severe-frequency (OR 2.73) and very high-frequency (HbA1c> 9%) (OR 2.41) were also risk factors. The same independent effects of severe hypoglycemia on poor neurocognitive and educational outcomes have been observed in the past, but organized monitoring and harsher self-management led to higher functional performance [20]. Various interacting factors affect the academic performance of children with T1DM, including socioeconomic status, parental education, school environment, psychological well-being, and overall disease burden. Hence, the relationship between hypoglycemia frequency and scholastic outcomes in the study should be viewed as an association rather than a causal relationship. Although a significant negative relationship was established, it is not possible to infer temporal or causal relationships due to the cross-sectional nature of the research. The observed findings could also be due to residual confounding by unmeasured variables. Therefore, despite the apparent significance of recurrent hypoglycemia as a correlate of poor academic performance, longitudinal and interventional research are necessary to establish causality.

This study has several limitations that should be considered. First, its cross-sectional design limits the ability to establish temporal or causal relationships between hypoglycemia and academic performance. Second, the study was conducted at a single tertiary-care center, which may limit the generalizability of the findings to other populations and settings. Third, although standardized records were used, some variables, such as hypoglycemia frequency and classroom functioning, may be subject to reporting bias or recall inaccuracies. Additionally, important confounding factors including socioeconomic status, parental education, school environment, psychological stress, and learning support were not fully assessed, which may influence academic outcomes. Variability in teaching quality and assessment methods across schools could also affect performance measures.

## Conclusions

We conclude that recurrent hypoglycemia is significantly associated with poorer academic performance among children with T1DM, as evidenced by lower examination scores, reduced school attendance, and increased absenteeism, with a clear gradient showing worse outcomes at higher frequencies of hypoglycemic episodes. Hypoglycemia frequency demonstrated a stronger association with scholastic performance than overall glycemic control (HbA1c), suggesting that acute glycemic fluctuations may play a more relevant role in day-to-day cognitive functioning; however, these findings should be interpreted cautiously, as the cross-sectional design does not establish causality and academic performance is influenced by multiple confounding factors including socioeconomic status, educational support, and psychological well-being.
